# GCRV-II Triggers B and T Lymphocyte Apoptosis via Mitochondrial ROS Pathway

**DOI:** 10.3390/v17070930

**Published:** 2025-06-30

**Authors:** Jie Wang, Wen-Jing Dong, Chang-Song Wu, Tian-Tian Tian, Xu-Jie Zhang, Yong-An Zhang

**Affiliations:** 1 National Key Laboratory of Agricultural Microbiology, Hubei Hongshan Laboratory, Engineering Research Center of Green Development for Conventional Aquatic Biological Industry in the Yangtze River Economic Belt, Ministry of Education, College of Fisheries, Huazhong Agricultural University, Wuhan 430070, China; wangjie@mail.hzau.edu.cn (J.W.); hdwj2024@webmail.hzau.edu.cn (W.-J.D.); wcs@mail.hzau.edu.cn (C.-S.W.); ttt@webmail.hzau.edu.cn (T.-T.T.); 2 Shenzhen Institute of Nutrition and Health, Huazhong Agricultural University, Wuhan 430070, China; 3 Shenzhen Branch, Guangdong Laboratory for Lingnan Modern Agriculture, Genome Analysis Laboratory of the Ministry of Agriculture, Agricultural Genomics Institute at Shenzhen, Chinese Academy of Agricultural Sciences, Shenzhen 518120, China

**Keywords:** GCRV-II, apoptosis, Caspase-3, B and T lymphocytes, reactive oxygen species

## Abstract

Grass carp reovirus (GCRV), particularly the highly prevalent genotype II (GCRV-II), is known to infect peripheral blood leukocytes (PBLs) of grass carp. However, it is unclear whether GCRV-II can induce apoptosis in bystander lymphocytes within infected PBLs. Here, we have shown that GCRV-II infection induces apoptosis via the mitochondria-dependent caspase-3 pathway in infected PBLs. GCRV-II infection was also found to induce a significant increase in reactive oxygen species (ROS) accumulation in leukocytes and lymphocytes, accompanied by increased apoptosis in IgM^+^ B and CD4^+^ T lymphocyte subsets. Further studies have demonstrated that the targeted inhibition of mitochondrial ROS production can effectively attenuate apoptosis in neighboring B and T lymphocytes within infected PBLs, suggesting that GCRV-II-induced pro-apoptotic effects on bystander lymphocytes largely require the involvement of the mitochondrial-dependent ROS pathway. Taken together, our study reveals the underlying mechanism by which GCRV-II induces apoptosis in bystander B and T lymphocytes through ROS production, providing new insights into understanding the virus-induced pro-apoptotic mechanism in specific immune cells and a potential strategy for viral immune escape.

## 1. Introduction

The severe hemorrhagic disease caused by grass carp reovirus (GCRV) in grass carp (*Ctenopharyngodon idella*) fry, with mortality rates of up to 80% per year, has resulted in significant losses for the aquaculture industry [1]. To date, several GCRV isolates have been identified from diseased grass carp and can be classified into three main genotypic groups, including GCRV-I, GCRV-II, and GCRV-III [1]. Of these, GCRV-II is the most widespread and virulent strain, typically associated with a short latent period, causing more severe hemorrhagic disease and higher mortality [1,2,3]. Phylogenetically, all three subtypes of GCRV belong to the *Spinareoviridae* family, classified in the *Aquareovirus* genus, whose members consist of 11 double-stranded RNA genome segments encoding both seven structural and six non-structural proteins of the virus [1,3,4]. VP4, VP35, and VP56 are the three major outer capsid proteins that are encoded by the GCRV-II segment 6, 11, and 7 genes and are characteristic of the signature proteins used to detect the presence of virus particles [1,3,5]. A comprehensive understanding of viral infection mechanisms in different cell types, as well as virus-induced cellular processes and immune responses, is critical to elucidate viral invasion and evasion. Previous studies have demonstrated that GCRV infection induces endoplasmic reticulum (ER) stress and oxidative stress, leading to DNA fragmentation and apoptosis in multiple cell lines [6,7,8,9]. Emerging evidence suggests that GCRV-II exhibits broad infectivity, not only in susceptible cell lines but also in specific immune cells, including peripheral blood leukocytes (PBLs) [4] and monocytes/macrophages (Mo/Mφs) [10]. Interestingly, PBLs serve as the primary executors of cellular immune defense during viral infection and are also a major target cell for GCRV-II invasion [4]. However, it remains unclear whether GCRV-II-infected PBLs would undergo oxidative stress and apoptosis by similar or different cellular mechanisms to those observed in established cell lines.

Apoptosis is a programmed cell death process that is employed by multicellular organisms to maintain cellular homeostasis and combat viruses. Generally, virus infection activates nucleic acid-mediated antiviral responses along with a variety of ER stress [11], mitochondrial dysfunction [12], and oxidative stress [13] that directly or indirectly influence the induction of apoptosis. Nevertheless, apoptosis and necrosis in infected cells can also facilitate virus propagation while evading immune recognition, which benefits the prolongation of the viral life cycle [6,7,14,15]. In virus-infected cells, apoptosis can be initiated by two main proposed mechanisms: (i) the death receptor-mediated extrinsic pathway, and (ii) the mitochondria-mediated intrinsic pathway [15,16]. The death receptor pathway begins after activation of the tumor necrosis factor (TNF) family of proteins, including Fas, activated by the ligand FasL, and TNF-related apoptosis inducing ligand (TRAIL) receptors 1 and 2 activated by TRAIL, as well as TNF receptor 1 (TNFR1) activated by TNF [17]. In the intrinsic apoptotic pathway, proapoptotic stimuli such as nitric oxide (NO) and reactive oxygen species (ROS) can directly induce mitochondrial outer membrane permeabilization [18,19]. Mitochondrial permeabilization (MMP) permits the release of mitochondrial intermembrane space proteins, such as cytochrome c (Cytc), into the cytosol. Cytc interacts with the apoptotic release factor (Apaf1) and activates the intrinsic apoptosis initiator caspase-9 and the downstream effector caspases 3 and 7. Although these different apoptotic pathways are well characterized in viral infections, their roles and relative contributions in specific immune cells (e.g., B and T cells) during GCRV infection are not well understood. Notably, emerging studies have demonstrated that viruses induce apoptosis in immune cells through one or both of the apoptosis pathways. For example, Epstein–Barr virus (EBV) upregulates Fas expression in infected B cells [20], while human T-cell leukemia virus types 1 (HTLV-1) induces T-cell apoptosis via the ROS pathway [21]. In human immunodeficiency virus (HIV) infection, ROS-induced mitochondrial dysfunction can drive DNA damage and apoptosis in infected T cells and B cells [22,23,24]. In addition, monocyte-derived ROS can also induce DNA damage in neighboring lymphocytes, leading to apoptosis of CD4^+^ and CD8^+^ T cells and a poor immune response to SARS-CoV-2 infection [25,26].

ROS are generated as by-products of oxidative phosphorylation (OXPHOS) and act as a crucial signaling molecule in cell proliferation and survival. Mitochondria play a critical role in energy production within the cell and are also involved in ROS production. Indeed, many viruses have evolved strategies to interfere with mitochondria and induce intracellular ROS production, thereby supporting their replication and life cycle [18]. For example, hepatitis B virus X protein (HBx) directly interacts with voltage-dependent anion channel (VDAC) 3, which is part of the mitochondrial permeability transition pore, leading to MMP depolarization and ROS production [27]. Hepatitis C virus (HCV) core, E1, E2, and NS3 proteins have been shown to bind to mitochondria and inhibit electron transfer from respiratory complex I to the next electron carrier, resulting in MMP depolarization and ROS production [28]. ROS production as a result of MMP depolarization has also been documented during HIV infection, with the HIV Tat protein directly inducing mitochondrial membrane permeabilization [29]. Notably, several viruses can manipulate ROS-mediated apoptosis by the induction of ER and oxidative stress [30], as well as DNA damage [26] and mitochondrial ROS (mtROS) production [13,18,31]. However, the specific inhibition of ROS with an mtROS scavenger (e.g., Mito-TEMPO) diminished virus replication, apoptosis, and mitochondrial permeability [31]. These studies have shown that viruses have evolved strategies to exploit ROS production to promote their own replication, while inducing mitochondrial damage and apoptosis as the viruses exit the host cell and release their progeny [13,18].

In this study, we demonstrated that GCRV-II infection of PBLs triggers mitochondrial dysfunction and induces apoptosis. We have also revealed that the mitochondrial ROS pathway is involved in apoptosis, as target inhibition of mtROS production reduces the apoptotic frequency in bystander IgM^+^ B and CD4^+^ T lymphocytes within infected PBLs.

## 2. Materials and Methods

### 2.1. Animals

Healthy grass carp (100–200 g for leukocyte isolation) were purchased from Xiantao Hatchery (Hubei, China) and maintained in aquarium tanks with a water control system. All the fish were daily fed with commercial pellets at a rate of 1% biomass and acclimatized to laboratory conditions for two weeks prior to the experiments. All animal experiments were conducted in accordance with the Guide for the Care and Use of Laboratory Animals of the Ministry of Science and Technology of China and approved by the Animal Ethics Committee of Huazhong Agricultural University (HZAU), and protocols were approved by license (HZAUFI-2024-0035). Every effort was made to minimize animal suffering.

### 2.2. Cell Isolation and Culture

High-purity leukocytes were isolated using discontinuous Percoll (Cat. #17089109, GE Healthcare, Chicago, IL, USA) density gradients and counted using the Cellometer Auto1000 (Nexcelom) according to our previous methods [32,33]. Firstly, blood was collected from the caudal vein of grass carp and immediately diluted with 5-fold volume of Roswell Park Memorial Institute incomplete medium (RPMI-1640, Cat. #11879020, Gibco, Waltham, MA, USA). The medium was supplemented with 25 U/mL heparin (Cat. #PHR8927, Merck, Darmstadt, Germany), 100 U/mL penicillin, and 100 μg/mL streptomycin (Cat. #15070063, Gibco, Waltham, MA, USA). PBLs were isolated with 51/34% discontinuous Percoll and centrifuged at 450× *g* for 30 min. The leukocytes lying at the interface were collected and washed three times with RPMI-1640 medium supplemented with 10 U/mL heparin. All isolated leukocytes were either resuspended in FACS buffer (PBS containing 2% fetal bovine serum (FBS, Cat. # 10099-141, Gibco, Waltham, MA, USA)) for the flow cytometry or cultured in the RPMI-1640 medium supplemented with 10% FBS, 100 U/mL penicillin, and 100 μg/mL streptomycin at a density of 1 × 10^6^ cells/mL at 28 °C with 5% CO_2_.

### 2.3. Viral Infection

The GCRV-II strain, GCRV-AH528, was maintained in our laboratory for viral infection. In brief, the isolated PBLs were adjusted to 1 × 10^6^ cells/mL, seeded in 24-well plates, and infected with GCRV-AH528 at a multiplicity of infection (MOI) of 1. The cells were then cultured in FBS-free RPMI-1640 medium for 4 h, as described in previous studies [4,5,10]. After that, the medium was then replaced with RPMI-1640 medium containing 5% FBS. The cells were collected at the indicated times after GCRV-II infection.

### 2.4. Apoptosis Detection

PBLs were prepared in 24-well plates and infected with GCRV-II (AH528) at an MOI of 1 [4,10] for 24, 36, and 48 h. After three washes with PBS, the cells were harvested and resuspended in binding buffer. They were then stained with an Annexin V-APC/PI Apoptosis Detection Kit (Cat. #40302ES50, Yeasen, Shanghai, China) for 15 min at room temperature, after which they were analyzed by flow cytometry (BD FACSVerse, Franklin Lakes, NJ, USA). To examine the apoptotic frequency of IgM^+^ B and CD4^+^ T lymphocytes after 36 h exposure to GCRV-II, the harvested PBLs (1 × 10^6^ cells) were resuspended in FACS buffer and then stained with FITC-labeled mouse anti-grass carp IgM mAb (1 μg/mL) [33] and rat anti-carp CD4 (Cat. #CAC-NIH-NA-01, COMSO-BIO, Tokyo, Japan, 1 μg/mL) [34,35] or stained with FITC-mouse IgG1 isotype Ab (Cat. #400103, BioLegend, San Diego, CA, USA, 1 μg/mL) and rat IgG1 isotype Ab (Cat. #401901, BioLegend, San Diego, CA, USA, 1 μg/mL) on ice for 45 min. The cells were then washed twice with FACS buffer and stained with PE-goat anti-rat IgG (Cat. #405406, BioLegend, San Diego, CA, USA, 1 μg/mL) for another 45 min, followed by the Annexin V-APC staining. Data on flow cytometry were analyzed using FlowJo (v10.4.1). To further detect the occurrence of apoptosis, PBLs infected with or without GCRV-II were cultured in confocal dishes for 48 h. After washing with PBS, the cells were stained with 1 µg/mL Hoechst 33258 (Cat. #C1017, Beyotime, Shanghai, China) for 10 min at room temperature. Imaging was performed using an inverted confocal microscope (Nikon N-STORM, Tokyo, Japan).

### 2.5. Mitochondrial Membrane Potential Measurement

Mitochondrial membrane potential (MMP) was measured using JC-1 (Cat. #C2006, Beyotime, Shanghai, China) staining as an indicator of mitochondrial depolarization. In brief, PBLs were incubated with or without GCRV-II for 12 h, 24 h, and 36 h and then stained with JC-1 (5 μM) for 20 min at room temperature according to the manufacturer’s instructions, followed by flow cytometry analysis. Data on flow cytometry were analyzed using FlowJo (v10.4.1).

### 2.6. Determination of Reactive Oxygen Species (ROS) and Mitochondrial ROS

To detect intracellular ROS production, GCRV-infected PBLs at 24 h were harvested and loaded with 2,7-dichlorofluorescein diacetate (DCFH-DA) probe (10 μM, Cat. #S0034S, Beyotime, Shanghai, China) for 20 min according to the manufacturer’s instructions. To detect ROS levels in IgM^+^ B and CD4^+^ T lymphocytes, PBLs were, respectively, stained with mouse anti-grass carp IgM mAb (1 μg/mL) or rat anti-carp CD4 (Cat. #CAC-NIH-NA-01, COMSO-BIO, Tokyo, Japan, 1 μg/mL) for 45 min, followed by staining with APC goat anti-mouse IgG (Cat. #405308, Biolegend, San Diego, CA, USA, 1 μg/mL) or APC goat anti-rat IgG (Cat. #405407, Biolegend, San Diego, CA, USA, 1 μg/mL) for another 45 min on ice. After that, cells were loaded with DCFH-DA for 20 min and then analyzed by flow cytometry. For detection of mitochondrial ROS (mtROS) levels, PBLs were stained with MitoSOX Red (2.5 μM, Cat. #HY-D1055, MCE, Monmouth Junction, NJ, USA) for 15 min and then washed with FACS buffer twice, followed by flow cytometry (BD FACSVerse, Franklin Lakes, NJ, USA). Data on flow cytometry were analyzed using FlowJo (v10.4.1).

### 2.7. Inhibitor Treatment

To investigate whether mtROS production is associated with cell apoptosis, a specific mtROS scavenger, Mito-TEMPO (15 µM) [36,37], was used to inhibit mtROS production in leukocytes and lymphocytes. After a 2 h pre-treatment, PBLs were infected with GCRV-II for 24 h to assess mtROS levels. Subsequently, at 36 h post infection, caspase-3 protein expression and the apoptotic rates of IgM^+^ B and CD4^+^ T lymphocytes were analyzed by Western blot and flow cytometry, respectively.

### 2.8. Transmission Electron Microscopy

Transmission electron microscopy (TEM) was used to observe the morphology and ultrastructure of PBLs with or without exposure to GCRV-II, as described previously [32,38]. Briefly, all the isolated cells were fixed overnight in 2.5% glutaraldehyde and post-fixed with 1% osmium tetroxide for 1 h. After stepwise acetone dehydration, cells were embedded in epoxy resin and polymerized at 80 °C for 72 h. Then, the embedded cells were sectioned to 50~70 nm thickness and stained with 2% uranyl acetate and lead citrate before being observed with a transmission electron microscope (Hitachi HT7700, Tokyo, Japan).

### 2.9. RNA-seq

PBLs isolated from triplicate fish samples from each group are incubated with or without GCRV-II for 24 h. The RNA-Seq libraries were generated as described previously [39]. In brief, the total RNA from each sample was extracted using the RNeasy Mini Kit with DNase I (Cat. #74104, Qiagen, Hilden, Germany). The concentration and purity of RNA were measured by NanoDrop 2000 (Thermo Fisher Scientific, Waltham, MA, USA). The integrity of RNA was measured using the 2100 Bioanalyzer (Agilent, Santa Clara, CA, USA) and the RNA integrity number (RIN) value ≥ 8. Then, the total RNA was sent to Shanghai Personalbio Technology (Shanghai, China) for next-generation sequencing (NGS) using the Illumina Xten genomic sequencing platform. The sequenced raw reads (150 bp paired-end reads) were first filtered to remove the adaptors and low-quality reads using Trimmomatic. Clean reads were aligned to the *Ctenopharyngodon idella* genome by HISAT2. Gene expression level was quantified using Cufflinks, and the differentially expressed genes were determined using DESeq2 [40]. To understand the potential functions of genes, we annotated the genes of *Ctenopharyngodon idella* by mapping to public databases, including the NCBI nonredundant protein database, Gene Ontology (GO), Swiss-Prot/UniProt, and Kyoto Encyclopedia of Genes and Genomes (KEGG). Genes were considered as differentially expressed genes (DEGs) if the *p*-value < 0.01 and |log_2_FC| > 1. All raw data in this study are available in the NCBI Sequence Read Archive Database (http://www.ncbi.nlm.nih.gov/Traces/sra/) (2 May 2025) under accession number PRJNA1258200. For gene set enrichment analysis (GSEA), RNA-seq data from GCRV-II and the control group were analyzed by GSEA (http://software.broadinstitute.org/gsea/downloads.jsp) (2 May 2025).

### 2.10. Semi-Quantitative and Quantitative Real-Time PCR

For semi-quantitative real-time PCR, the Trizol-extracted total RNA (1 µg) was used for cDNA synthesis with a reverse transcription kit (Cat. #RR047B, Takara, Kyoto, Japan). The synthesized cDNA was diluted five times and then used as the template for PCR amplification. Semi-quantitative PCR was performed using a 20 µL reaction system, containing 10 µL of Taq Master Mix (Cat. #P112, Vazyme, Nanjing, China), 8 µL of RNase-free water, 1 µL of diluted cDNA (0.2 µg), and 0.5 µL of viral gene-specific primer (10 µM). PCR amplification procedures were as follows: 95 °C for 5 min; 95 °C for 30 s, 55 °C for 30 s, and 68 °C for 2 min, for 35 cycles. Then they were extended at 72 °C for 10 min. The PCR products were then subjected to gel electrophoresis and photographed. For quantitative real-time PCR (qPCR), the reaction volume contained 10 μL ChamQ SYBR Color qPCR Master Mix (Cat. #Q411-02, Vazyme, Nanjing, China), 0.5 μL of each primer, and 1 μL diluted cDNA. The amplification procedures were 95 °C for 5 min followed by 40 cycles at 95 °C for 10 s and 60 °C for 30 s, and then a melt curve was acquired from 65 °C to 95 °C using a CFX ConnectTM Real-Time System (Bio-Rad, Hercules, CA, USA). Each sample was performed in triplicate. Gene expression levels were quantified using the 2^−ΔΔCt^ method [41], with *ef1* as the internal control. The primers used for the PCR assay are listed in Table 1.

### 2.11. SDS-PAGE and Western Blot

The purified proteins and cell lysates were collected and boiled in the SDS-PAGE loading buffer (Cat. #BL502B, Biosharp, Hefei, China) at 95 °C for 10 min and then electrophoresed in 4–15% precast polyacrylamide gels (Cat. #4561085, Bio-Rad, Hercules, CA, USA). The gels were transferred onto nitrocellulose membranes (Cat. #1020145, Bio-Rad, Hercules, CA, USA) using a semi-dry Trans-Blot (Bio-Rad). The membranes were blocked with 5% skim milk and then incubated with rabbit anti-VP4 pAb (developed in our lab) and mouse anti-β-actin mAb (Cat. #66009-1-Ig, Proteintech, Chicago, IL, USA), or with Caspase-3 rabbit mAb (Cat. #A19664, ABclonal, Wuhan, China) and α-Tubulin mouse mAb (Cat. # 66031-1-Ig, Proteintech, Chicago, IL, USA), followed by incubation with HRP-goat anti-rabbit IgG (Cat. #AS063, ABclonal, Wuhan, China) or HRP-goat anti-mouse IgG (Cat. #AS064, ABclonal, Wuhan, China) secondary Abs. Thereafter, the membranes were incubated with Clarity Western ECL Substrate (Cat. #1705060, Bio-Rad, Hercules, CA, USA) and scanned using the Amersham Imager 600 (GE Healthcare, Chicago, IL, USA). The band density was analyzed using ImageJ software (v1.46r).

### 2.12. Statistical Analysis

All images shown in this study are representative of two or three independent experiments, and all bar charts show the mean ± SEM derived from at least three independent repeats. Unpaired *t*-test and one-way ANOVA followed by Tukey’s multiple comparison test were performed in Prism 8 for analysis of differences between groups. A significant difference was accepted at *p* < 0.05 (*, *p* < 0.05, **, *p* < 0.01, ***, *p* < 0.001, ****, *p* < 0.0001).

## 3. Results

### 3.1. GCRV-II Infects PBLs and Induces Mitochondrial Dysfunction

**Figure 1:**
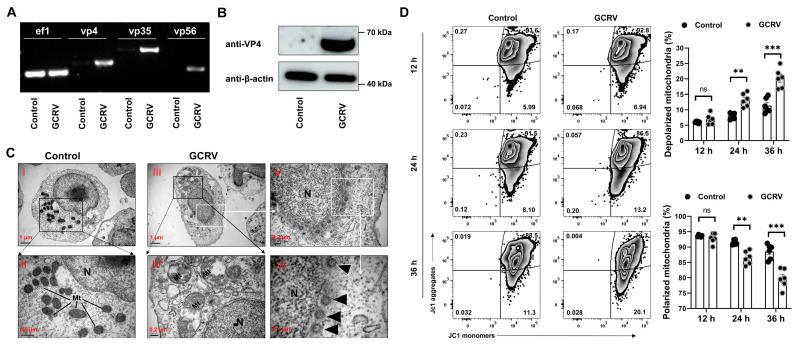
GCRV-II infects PBLs and induces mitochondrial dysfunction. (**A**) Gene expression of *vp4*, *vp35*, and *vp56* in PBLs at 24 h post infection. *ef1* was employed as an internal control. (**B**) Protein expression of VP4, with β-actin as an internal reference, in PBLs at 24 h post infection. (**C**) TEM observation of the morphology and ultrastructure of PBLs and virus particles at 24 h post infection (N, nucleus; Mt, mitochondria; black arrows indicate virus particles). Scale bar, 1 µm (Images **I**,**III**), 0.5 µm (Image **II**), 0.2 µm (Images **IV**,**V**), and 0.1 µm (Image **VI**). (**D**) Mitochondrial permeabilization of PBLs detected by JC1 at 12–36 h post infection. n = 6. Data acquisition required two or three independent tests (mean ± SEM). ** *p* < 0.01, *** *p* < 0.001. PBLs, peripheral blood leukocytes; TEM, transmission electron microscope.

Previous studies have shown that GCRV-II is capable of infecting PBLs and Mo/Mφs in vitro [4,10]. In addition, the GCRV-II outer capsid protein VP4 has been demonstrated to induce MMP depolarization, resulting in mitochondrial damage and apoptosis in *Ctenopharyngodon idella* kidney (CIK) cells [9]. To determine the potential of GCRV-II to infect PBLs and induce mitochondrial dysfunction, the PBLs were isolated and cultured in serum-free medium and exposed to GCRV-II for 4 h to facilitate viral invasion, after which they were cultured in an FBS medium for a further 20 h. Our data show that GCRV-II is able to infect PBLs and complete viral gene (e.g., *vp4*, *vp35*, and *vp56*) replication and viral VP4 protein translation (Figure 1A,B). Using transmission electron microscopy (TEM), we observed that the viral particles were located in the cytoplasm close to the nucleus (Images V and VI in Figure 1C). Notably, we also observed obvious mitochondrial swelling and damage in infected PBLs, accompanied by a marked reduction in their number (Images II and IV in Figure 1C). To further validate the occurrence of mitochondrial dysfunction in the infected PBLs, we assessed the mitochondrial membrane potential using the JC1 probe at 12–36 h post infection. The results showed pronounced mitochondrial dysfunction in infected PBLs, as evidenced by a significantly increased frequency of unhealthy or depolarized mitochondria and a decreased ratio of polarized mitochondria, at 24 and 36 h rather than 12 h post infection (Figure 1D). These results collectively suggest that GCRV-II can directly invade PBLs and induce mitochondrial dysfunction through mitochondrial depolarization.

### 3.2. GCRV-II Triggers Mitochondria-Dependent Apoptosis Pathway in PBLs

**Figure 2:**
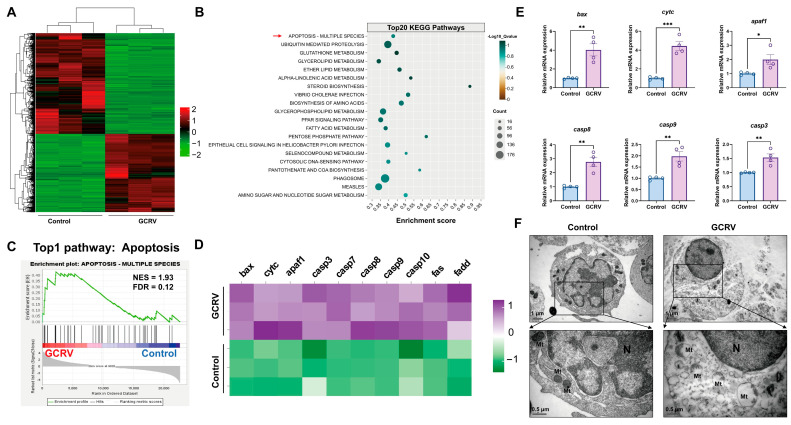
GCRV-II triggers mitochondria-dependent apoptosis pathway in PBLs. (**A**) Heatmap analysis of phenotypic DEGs between GCRV and control group (data adjusted *p* < 0.01, |log_2_FC| > 1; red, upregulated genes; green, downregulated genes). *n* = 3. (**B**) Top 20 KEGG pathways enriched for GCRV group by GSEA. The red arrow indicates the selected pathways in Figure 2C. (**C**) Significantly enriched gene sets related to apoptosis in GCRV group at 36 h post infection (NES, normalized enrichment score; FDR, false discovery rate). (**D**) Heatmap analysis of phenotypic DEGs related to apoptosis pathway (data adjusted *p* < 0.01, |log_2_FC| > 1; violet, upregulated genes; green, downregulated genes). n = 3. (**E**) Relative mRNA levels of the mitochondria-mediated proapoptotic genes in PBLs at 36 h post infection. n = 4. (**F**) TEM observation of the morphology and ultrastructure of PBLs at 36 h post infection (N, nucleus; Mt, mitochondria). Scale bar, 1 µm (original), 0.5 µm (enlarged). Data acquisition required two or three independent tests (mean ± SEM). * *p* < 0.05, ** *p* < 0.01, *** *p* < 0.001. PBLs, peripheral blood leukocytes; DEGs, differentially expressed genes; GSEA, gene-set enrichment analysis; TEM, transmission electron microscope.

Previous studies have shown that virus-induced mitochondrial depolarization is a clear indicator associated with early apoptosis in infected cells [18,19,30]. To investigate whether GCRV-II infection has an impact on cell survival and cellular immune response, we performed RNA sequencing (RNA-seq) and gene set enrichment analysis (GSEA) in PBLs with or without virus exposure. As shown in Figure 2, the GCRV group showed a completely different gene expression pattern compared to the control group (Figure 2A). Among the top 20 enriched KEGG pathways in the GCRV group, the apoptosis pathway ranked first (Figure 2B,C), with significant upregulation of pro-apoptotic genes (e.g., *bax*, *cytc*, *apaf1*, *casp3*, *casp7*, *casp8*, *casp9*, *casp10*, *fas*, and *fadd*) (Figure 2D). The qPCR data further confirmed the expression profile (*bax*, *cytc*, *apaf1*, *casp3*, *casp8*, and *casp9*) consistent with mitochondria-mediated intrinsic apoptosis in PBLs (Figure 2E). In addition, the infected PBLs showed typical apoptotic symptoms such as cytoplasmic vacuolization, cell rounding, chromatin condensation, and membrane blebbing, accompanied by obvious mitochondrial damage (Figure 2F). Notably, PBLs in the GCRV group have also shown an obvious antiviral response characterized by the upregulation of interferon-related genes (e.g., *IFNγ*, *IFNα*, and *IFNβ*) (Figure S1B,C), suggesting that viral infection could also activate nucleic acid-mediated cellular defenses through antiviral signaling pathways including TLR-like receptor, JAK-STAT, and cGAS-STING (Figure S1D). The transcriptome and qPCR analysis collectively indicated that GCRV-II infection initiates a mitochondria-dependent apoptosis pathway in infected PBLs.

### 3.3. GCRV-II Induces Cell Apoptosis and Necrosis Through Caspase-3 Activation

**Figure 3:**
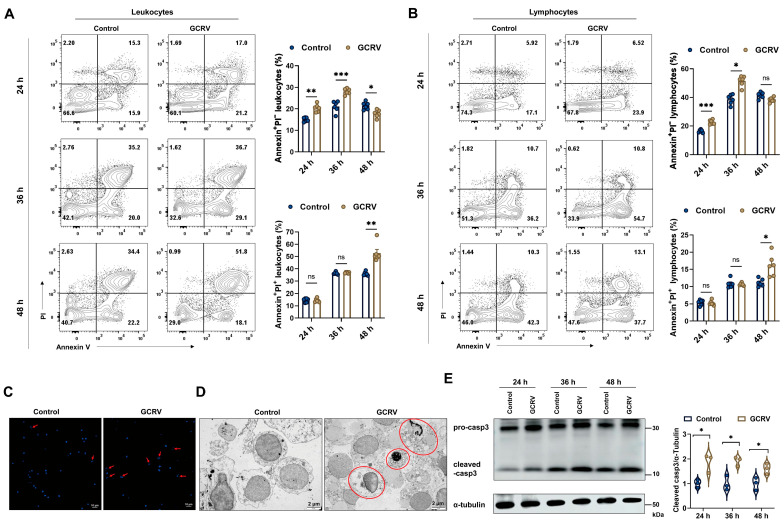
GCRV-II induces cell apoptosis and necrosis through caspase-3 activation. (**A**,**B**) The respective frequencies of Annexin^+^PI^-^ and/or Annexin^+^PI^+^ cells in leukocytes (**A**) and lymphocytes (**B**) at 24, 36, and 48 h post infection. n = 6. (**C**) Hoechst staining of PBLs at 48 h post infection. Red arrows point to typical apoptotic symptoms of nuclear fragmentation. Scale bar, 10 µm. (**D**) TEM observation of morphology and ultrastructure in the late apoptotic and necrotic cells at 48 h post infection. Red circles indicate the late apoptotic and necrotic cells with chromatin condensation, nuclear fragmentation, and membrane blebbing. Scale bar, 2 µm. (**E**) Protein levels of cleaved casp3 in PBLs at 24, 36, and 48 h post infection. n = 3. Data acquisition required two or three independent tests (mean ± SEM). * *p* < 0.05, ** *p* < 0.01, *** *p* < 0.001. PBLs, peripheral blood leukocytes; TEM, transmission electron microscope; casp3, caspase-3.

To assess the dynamic changes in the frequency of apoptotic cells, we employed the Annexin V/PI staining assay followed by flow cytometry analysis. In early apoptotic cells, phosphatidylserine (PS) exposure to the outer plasma membrane leaflet could be detected by Annexin V staining, whereas propidium iodide (PI) counterstaining could further distinguish the late apoptotic and necrotic cells. In our study, GCRV-II infection significantly induced apoptosis and necrosis in both blood leukocytes and lymphocytes from 24 to 48 h (Figure 3A,B). However, early apoptosis was predominant in the initial infection (24–36 h), whereas late apoptosis and necrosis were more pronounced in the late phase within 48 h (Figure 3A,B). At 48 h post infection, GCRV-II-infected PBLs exhibited typical apoptotic and necrotic features, including cytoplasmic shrinkage, nuclear chromatin condensation, DNA fragmentation, and membrane blebbing (Figure 3C,D). On the other hand, infected PBLs showed higher protein levels of cleaved caspase-3 at indicated time points (24, 36, and 48 h) after infection (Figure 3E), suggesting that virus-induced apoptosis in PBLs was associated with the activation of the caspase-3 pathway.

### 3.4. GCRV-II Induces ROS Production and Apoptosis in IgM^+^ B and CD4^+^T Lymphocytes

**Figure 4:**
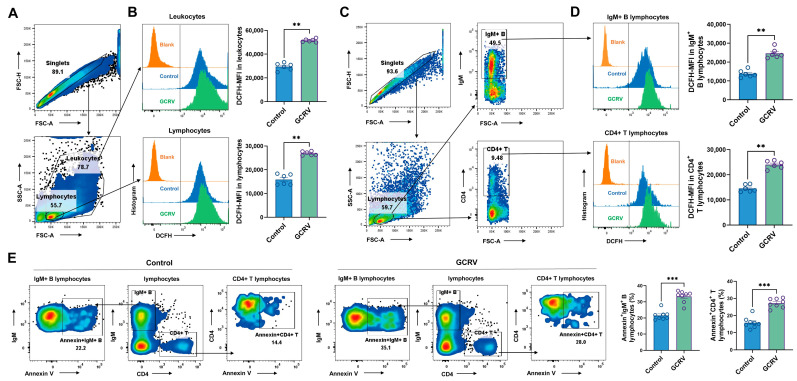
GCRV-II induces ROS production and apoptosis in IgM^+^ B and CD4^+^T lymphocytes. (**A**) Flow cytometry gating strategy of leukocytes and lymphocytes in PBLs. (**B**) ROS levels (DCF-MFI) in leukocytes (top panel) and lymphocytes (bottom panel) at 24 h post infection. n = 6. (**C**) Flow cytometry gating strategy of IgM^+^ B and CD4^+^ T lymphocytes. (**D**) ROS levels (DCF-MFI) of IgM^+^ B cells (top panel) and CD4^+^ T lymphocytes (bottom panel) at 24 h post infection. n = 6. Blank indicates blank control staining. (**E**) The respective frequencies of apoptotic IgM^+^ B and CD4^+^ T lymphocytes stained with Annexin V at 36 h post infection. n = 8. Data acquisition required two or three independent tests (mean ± SEM). ** *p* < 0.01, *** *p* < 0.001. PBLs, peripheral blood leukocytes; ROS, reactive oxygen species.

Since increased ROS can directly cause DNA damage, leading to apoptosis [13], we next analyzed the levels of intracellular ROS using the indicator dye DCFH-DA loaded in infected PBLs at different gating subpopulations (Figure 4A,C). As shown in Figure 4, GCRV-II infection could significantly promote intracellular ROS production in both leukocytes and lymphocytes at 24 h post infection (Figure 4B). More critically, we also found that ROS levels in gated IgM^+^ B and CD4^+^ T lymphocytes increased significantly at 24 h post infection (Figure 4C,D), suggesting that GCRV-II infection could potentially induce ROS accumulation in neighboring B and T lymphocytes within infected PBLs. To further investigate the ROS overload through which GCRV-II infection may induce apoptosis in IgM^+^ B and CD4^+^ T lymphocytes, PBLs with or without virus exposure were stained with anti-IgM or anti-CD4 mAbs and then counterstained with Annexin V. As our data showed, the frequency of apoptotic B and T lymphocytes was significantly increased in the GCRV-II-infected PBLs (Figure 4E), suggesting that ROS overproduction upon viral infection may be a potential inducer to provoke apoptosis in bystander B and T lymphocytes.

### 3.5. Inhibition of mtROS Production Attenuates GCRV-II-Induced Apoptosis in IgM^+^ B and CD4^+^ T Lymphocytes

**Figure 5:**
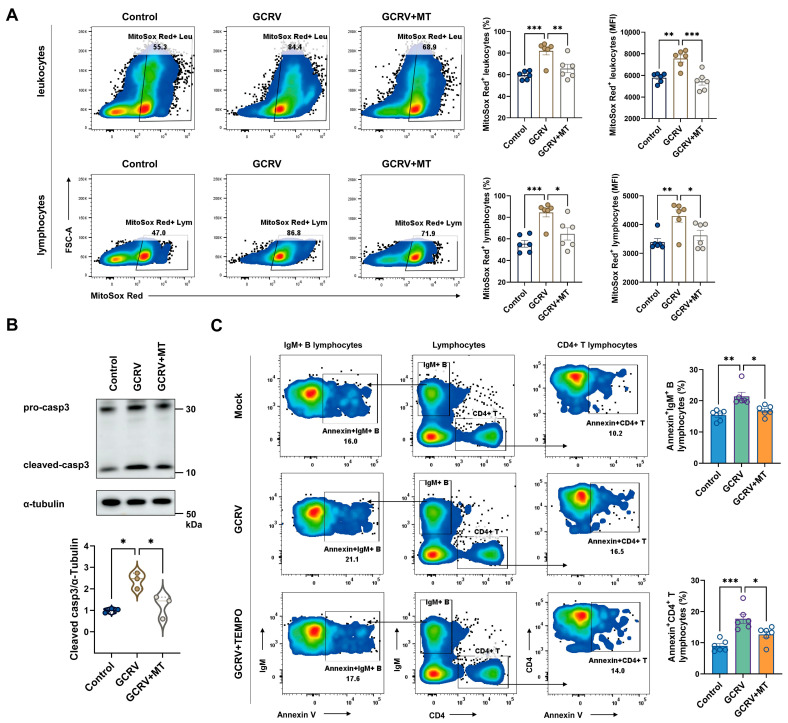
Inhibition of mtROS production attenuates GCRV-II-induced apoptosis in IgM^+^ B and CD4^+^T lymphocytes. (**A**) Frequency and fluorescence intensity of MitoSox Red^+^ cells in leukocytes (top panel) and lymphocytes (bottom panel) pretreated with or without MT at 24 h post infection. Blank indicates blank control staining. (**B**) Protein levels of cleaved caspase-3 in PBLs pretreated with or without MT at 36 h post infection. n = 3. (**C**) The respective frequencies of apoptotic IgM^+^ B and CD4^+^T lymphocytes stained with Annexin V in PBLs pretreated with or without MT at 36 h post infection. n = 6. Data acquisition required two or three independent tests (mean ± SEM). * *p* < 0.05, ** *p* < 0.01, *** *p* < 0.001. mtROS, mitochondria reactive oxygen species; PBLs, peripheral blood leukocytes; MT, Mito-TEMPO; casp3, caspase-3.

Mitochondria are known as the powerhouses of a cell; they are the most suitable targets of the ROS produced inside a cell [13]. To further investigate whether GCRV-II-induced ROS overproduction was associated with mitochondrial oxidative stress, we examined mitochondrial ROS (mtROS) levels using MitoSox Red staining in both leukocytes and lymphocytes at 24 h post infection. The significantly higher mtROS levels after GCRV-II infection could be observed by analyzing the frequency of MitoSox Red^+^ cells and the fluorescence intensity of MitoSox Red within the gated leukocyte and lymphocyte populations (Figure 5A). However, GCRV-II-induced mitochondrial ROS was effectively attenuated by pre-treatment with Mito-TEMPO (MT), a mitochondria-targeted antioxidant (Figure 5A). On the other hand, caspase-3-mediated apoptosis in PBLs was significantly enhanced by mtROS overload induced by GCRV-II infection, whereas it was attenuated by mtROS suppression followed by MT treatment (Figure 5B). Notably, we next analyzed apoptosis in gated lymphocytes and found that MT pretreatment can also reduce the apoptotic frequency in both IgM^+^ B and CD4^+^ T lymphocytes after exposure to GCRV-II (Figure 5C). Taken together, these results suggest that suppression of mtROS production in infected PBLs could ameliorate the GCRV-II-driven pro-apoptotic effects on bystander B and T lymphocytes.

## 4. Discussion

Viruses are obligate acellular parasites that hijack host cellular machineries for genome replication, protein translation, virion assembly, and virus particle release. For optimal outcome, viruses have evolved strategies to interfere with host antiviral defenses and subvert host cellular pathways for their own replication and dissemination [18]. Mitochondria-mediated oxidative stress and DNA damage typically occur in virus-infected cells [18,19]. In this study, we have found that GCRV-II can infect PBLs and induce mitochondrial dysfunction by decreasing mitochondrial membrane potential (MMP). Similarly, emerging studies have shown that GCRV-II major outer capsid protein VP4 caused loss of MMP, which allows a large amount of Ca^2+^ to enter mitochondria and leads to mitochondrial stress [9]. Notably, loss of MMP is associated with early apoptosis, which has been attributed to interactions between viral proteins and mitochondrial membranes [19]. For instance, HBV HBx protein directly interacts with VDAC3, an outer mitochondrial membrane porin and component of the mitochondrial permeability transition pore, leading to mitochondrial membrane conductance and MMP depolarization [27]. GCRV-II VP4 protein has also been reported to interact with VDAC2 and induce mitochondria damage and apoptosis [9]. As an evolutionarily conserved antiviral strategy across fish to mammals, apoptosis effectively limits viral propagation through selective removal of infected cells while initiating immune defenses via induction of antiviral cytokines [7,42]. However, beyond its role in the cellular antiviral response, induction of apoptosis has emerged as a common strategy employed by viruses to facilitate virion release and dissemination [9,10,15,18]. Recent studies have shown that GCRV was not only infectious in various cell lines such as CIK [7,9,43] and GCO (grass carp ovary) [5], but also in blood leukocytes [4] and Mo/Mφs [10]. In particular, GCRV infection can induce apoptosis and oxidative stress in both susceptible cell lines and specific immune cells such as monocytes/macrophages [6,7,10]. Similarly, in this study, the transcriptomic analysis, qPCR data, and immunoblotting results collectively showed that GCRV-II can induce apoptosis and necrosis in PBLs via the mitochondria-mediated caspase-3 pathway. This suggests that induction of leukocyte apoptosis may be a potential strategy by which GCRV-II can enhance its own spread, particularly in the absence of a cytopathic effect [10]. Leukocytes serve as the primary executors of the cellular immune response to viral infection and are also a major target cell for the invasion of Oyster herpesvirus (OsHV-1) [44], white spot syndrome virus (WSSV) [45], and GCRV-II [4]. OsHV-1 infection could trigger apoptosis of type II granulosa cells (analogous to fish leukocytes), facilitating viral dissemination and immune evasion [44]. Similarly, apoptosis of Mo/Mφs was clearly observed after GCRV-II infection, suggesting that induction of leukocyte apoptosis may be a potential strategy by which GCRV-II enhances its spread and immune escape [10]. Intriguingly, despite its pro-apoptotic effects, GCRV-II infection in leukocytes also elicits robust interferon responses, which is consistent with a previous study [4]. It was suggested that there exists a complex interplay between host defense and viral subversion. Emerging studies have also shown that Sendai virus (Sev) can trigger the RIG-I/IRF3-mediated mitochondrial apoptosis pathway [46,47]. Additionally, SARS-CoV-2 infection can activate IFNγ and TLR signaling pathways, which cause cell death via caspase-8, iNOS, and BAX/BAK in macrophages [48]. Collectively, these findings suggest that viruses may take advantage of stimulating apoptosis, either to induce the breakdown of infected cells via the cellular antiviral response, or to kill uninfected cells from the host immune system, thereby favoring viral dissemination.

Another interesting finding of our study is that apoptosis also occurred in bystander lymphocytes, including IgM^+^ B and CD4^+^T lymphocytes. Similar results have been observed in HIV and IAV infection: the HIV tat protein can directly induce DNA damage in B and T lymphocytes via mitochondrial dysfunction during HIV infection [22,24], while a proportion of lymphocyte subpopulations such as CD4^+^ T and CD19^+^ B cells can ultimately undergo apoptosis following exposure of peripheral blood mononuclear cells (PBMCs) to IAV [49]. In this study, the increased frequency of apoptosis was clearly observed in lymphocytes as well as B and T lymphocytes after exposure of PBLs to GCRV-II, suggesting that GCRV-II may potentially induce lymphopenia in infected PBLs, similar to that observed in HIV and IAV infection [24,49,50]. This phenomenon is also consistent with the concept proposed in previous studies that virus-induced apoptosis in specific immune cells, such as macrophages, B cells, and T cells, can enhance viral spread by facilitating immune evasion and systemic dissemination [10,21,24,49,50]. Notably, the percentage of IAV-infected lymphocytes was shown to be lower than the percentage of apoptotic lymphocytes, suggesting that direct effects of cell infection by the IAV could not fully account for the high level of cell death [50]. On the other hand, removal of Mo/Mφs after IAV exposure significantly reduced the percentage of lymphocytes that were apoptotic [49]. Further studies have shown that active synthesis and expression of the neuraminidase by abortively infected Mo/Mφs is required to induce lymphocyte death [50,51]. Collectively, these studies propose that a possible mechanism for lymphocyte apoptosis induced by viral infection may largely depend on the pro-apoptotic signals/molecules from neighboring infected cells, such as infected Mo/Mφs [50]. However, whether GCRV-II-induced lymphocyte apoptosis is dependent or independent of the pro-apoptotic signals/molecules from infected PBLs needs to be further investigated. In our study, it was noteworthy that the apoptotic B and T lymphocytes were typically accompanied by the elevated ROS levels, while inhibition of ROS production in infected PBLs can indeed decrease the apoptotic frequency in bystander B and T lymphocytes. This suggests that the induction and accumulation of ROS are primary events for inducing apoptosis, whether in leukocytes or lymphocytes.

Notably, mitochondria have been shown to play a central role in energy production and are a major source of ROS within a cell [13,19]. Interactions between viral proteins and mitochondrial membranes generally result in the overproduction of mitochondrial ROS (mtROS), leading to depolarization and increased permeability [24,27,28], and leakage of mitochondrial contents is associated with the initiation of the intrinsic apoptosis pathway [18,19,31]. As such, the induction of mtROS production has emerged as an alternative strategy employed by many viruses to promote their replication [18,19,31]. In this study, GCRV-II infection also induced mtROS production in both leukocytes and lymphocytes, probably as a consequence of mitochondrial dysfunction and cell apoptosis. Increased mtROS and altered mitochondrial membrane potential were clearly observed in PBLs at 24 h post infection, when damaged mitochondria and increased apoptosis were detected with an additional increase afterwards (24–36 h). Inhibition of mtROS also reduced the caspase-3-mediated apoptosis in infected PBLs, suggesting that GCRV-II-induced mitochondrial stress and mtROS overload further contribute to the apoptotic process in infected cells. On the other hand, inhibition of mtROS production in PBLs also reduced the apoptotic frequency in IgM^+^ B and CD4^+^ T lymphocyte subsets, suggesting that the increased ROS levels in GCRV-II-infected cells can further provoke apoptosis in bystander B and T lymphocytes. Similarly, SARS-CoV-2 virus has been shown to exploit ROS production in infected monocytes to provoke apoptosis in neighboring T lymphocytes, further disrupting host cellular immunity and favoring their spread and escape [25,26]. At the same time, we found that general ROS and mtROS levels increased in both leukocytes and lymphocytes after GCRV-II infection, specifically inhibiting mtROS significantly reduced apoptosis, whether in infected leukocytes or bystander lymphocytes. This suggests that, rather than other ROS sources (e.g., cytosolic or NADPH oxidase-derived ROS) [31], mitochondria-derived ROS may play a more critical role in inducing apoptosis in both leukocytes and lymphocytes. However, the extent to which GCRV-II-induced lymphocyte apoptosis depends on mtROS production needs to be investigated further. Overall, our findings reveal a potential pro-apoptotic mechanism whereby GCRV-II infection of PBLs induces apoptosis via the mitochondrial ROS pathway in bystander B and T lymphocytes.

## 5. Conclusions

In conclusion, our study demonstrates that GCRV-II infection induces mitochondrial stress and triggers mitochondrial-mediated intrinsic apoptosis via caspase-3 activation in infected PBLs. On the other hand, GCRV-II infection can also induce increased ROS levels in leukocytes and lymphocytes, which could lead to apoptosis in both IgM^+^ B and CD4^+^ T lymphocyte subsets. More critically, our study establishes that these GCRV-II-driven pro-apoptotic effects on bystander lymphocytes can be significantly attenuated through the targeted inhibition of mitochondrial ROS, suggesting that ROS production in infected cells could provoke apoptosis in bystander B and T lymphocytes. These findings reveal an underlying mechanism by which GCRV-II infection induces apoptosis in bystander B and T lymphocytes via the mitochondrial ROS pathway.

## Figures and Tables

**Table 1:** Primers used in this study for qPCR analysis.

Primers	Sequence 5′-3′	Tm (°C)	Product Size (bp)
Ef1-F	CGCCAGTGTTGCCTTCGT	58	99
Ef1-R	CGCTCAATCTTCCATCCCTT		
Bax-F	CATCTATGAGCGGGTTCGTC	57	94
Bax-R	TTTATGGCTGGGGTCACACA		
Cytc-F	TGCATACTTAAAGTCAGCCACATC	58	97
Cytc-R	TAAAGACATCCGAATCCAAACAAC		
Apaf1-F	AAGTTCTGGAGCCTGGACAC	56	106
Apaf1-R	AACTCAAGACCCCACAGCAC		
Caspase8-F	GGTAATCTGGTTGAAATCCGTG	56	98
Caspase8-R	CCTTGGCAGGCTTGAATGA		
Caspase9-F	CCTGGAGCAGTTCATGGTGT	57	212
Caspase9-R	ATGGCGTCCATCTGGTCATC		
Caspase3-F	CTGATGGGGCATCTGGACTG	58	147
Caspase3-R	GTTGGTTCATGCCTGTCGTG		
Vp4-F	CGAAAACCTACCAGTGGATAATG	60	135
Vp4-R	CCAGCTAGTACGCCGACGAC		
Vp35-F	CATGCCAGTCATATTTGATC	51	195
Vp35-R	TGGGAGGTTGTGGTAGAA		
Vp56-F	AGCAGGCTATTCATCACCAGT	57	107
Vp56-R	GCTCTAACACTCACCGTCTTTTC		

## Data Availability

RNA sequencing data (NCBI SRA PRJNA1258200) have been deposited at NCBI and are publicly available as of the date of publication. Any additional information required to reanalyze the data reported in this paper is available from the corresponding authors upon request.

## Supplementary Materials

The following supporting information can be downloaded at: https://www.mdpi.com/article/10.3390/v17070930/s1, Figures S1 and S2: Flow cytometry gating strategy and antiviral immune pathways.

## Abbreviations

The following abbreviations are used in this manuscript:

PBLs	Peripheral blood leukocytes
GCRV-II	Type II grass carp reovirus
ER	Endoplasmic reticulum
Mo/Mφs	Monocytes/macrophages
ROS	Reactive oxygen species
MMP	Mitochondrial permeabilization
mtROS	Mitochondrial reactive oxygen species
MOI	Multiplicity of infection
TEM	Transmission electron microscopy
GSEA	Gene set enrichment analysis

## References

[1] Li P.W., Zhang J., Chang M.X. (2023). Structure, function and immune evasion strategies of aquareoviruses, with focus on grass carp reovirus. Rev. Aquac..

[2] Jiang R., Zhang J., Liao Z.W., Zhu W.T., Su H., Zhang Y.A., Su J.G. (2023). Temperature-regulated type II grass carp reovirus establishes latent infection in *Ctenopharyngodon idella* brain. Virol. Sin..

[3] Zhu W.T., Qiao M.H., Hu M.D., Huo X.C., Zhang Y.A., Su J.G. (2023). Type II grass carp reovirus rapidly invades grass carp (*Ctenopharyngodon idella*) via nostril–olfactory system–brain axis, gill, and skin on head. Viruses.

[4] Yang L., Su J.G. (2021). Type II grass carp reovirus infects leukocytes but not erythrocytes and thrombocytes in grass carp (*Ctenopharyngodon idella*). Viruses.

[5] Jiang L., Liu A.Q., Zhang C., Zhang Y.A., Tu J.G. (2022). Hsp90 regulates GCRV-II proliferation by interacting with VP35 as its receptor and chaperone. J. Virol..

[6] Dai Y.L., Li Y.Q., Lin G., Zhang J.J., Jiang N., Liu W.Z., Meng Y., Zhou Y., Fan Y.D. (2022). Non-pathogenic grass carp reovirus infection leads to both apoptosis and autophagy in a grass carp cell line. Fish Shellfish Immunol..

[7] Jia R., Cao L.P., Du J.L., Liu Y.J., Wang J.H., Jeney G., Yin G.J. (2014). Grass carp reovirus induces apoptosis and oxidative stress in grass carp (*Ctenopharyngodon idellus*) kidney cell line. Virus Res..

[8] Liang B., Su J.G. (2019). Inducible nitric oxide synthase (*iNOS*) mediates vascular endothelial cell apoptosis in grass carp reovirus (GCRV)-induced hemorrhage. Int. J. Mol. Sci..

[9] Zhao K.W., Zhang Y.S., Yin Z.J., Tan L.B., Juario M., Zhang H.Y., Liu Y.L., Xu P.X., Zhang Q., Zhao G.A. (2025). GCRV-II major outer capsid protein VP4 promotes cell apoptosis by VDAC2-mediated calcium pathway facilitation. Int. J. Biol. Macromol..

[10] Xia N., Zhang Y.Q., Zhu W.T., Su J.G. (2024). GCRV-II invades monocytes/macrophages and induces macrophage polarization and apoptosis in tissues to facilitate viral replication and dissemination. J. Virol..

[11] Barry G., Fragkoudis R., Ferguson M.C., Lulla A., Merits A., Kohl A., Fazakerley J.K. (2010). Semliki forest virus-induced endoplasmic reticulum stress accelerates apoptotic death of mammalian cells. J. Virol..

[12] Yang S., Gorshkov K., Lee E.M., Xu M., Cheng Y.S., Sun N., Soheilian F., de Val N., Ming G.L., Song H.J. (2020). Zika virus-induced neuronal apoptosis via increased mitochondrial fragmentation. Front. Microbiol..

[13] Reshi M.L., Su Y.-C., Hong J.-R. (2014). RNA viruses: ROS-mediated cell death. Int. J. Cell Biol..

[14] Mayank A.K., Sharma S., Nailwal H., Lal S.K. (2015). Nucleoprotein of influenza A virus negatively impacts antiapoptotic protein API5 to enhance E2F1-dependent apoptosis and virus replication. Cell Death Dis..

[15] Ampomah P.B., Lim L.H.K. (2020). Influenza A virus-induced apoptosis and virus propagation. Apoptosis.

[16] Jorgensen I., Rayamajhi M., Miao E.A. (2017). Programmed cell death as a defence against infection. Nat. Rev. Immunol..

[17] Zhou X.C., Jiang W.B., Liu Z.S., Liu S., Liang X.Z. (2017). Virus infection and death receptor-mediated apoptosis. Viruses.

[18] Foo J., Bellot G., Pervaiz S., Alonso S. (2022). Mitochondria-mediated oxidative stress during viral infection. Trends Microbiol..

[19] Finlay B.B., Galluzzi L., Brenner C., Morselli E., Touat Z., Kroemer G. (2008). Viral control of mitochondrial apoptosis. PLoS Pathog..

[20] Incrocci R., Hussain S., Stone A., Bieging K., Alt L.A.C., Fay M.J., Swanson-Mungerson M. (2015). Epstein-barr virus latent membrane protein 2A (LMP2A)-mediated changes in Fas expression and Fas-dependent apoptosis: Role of Lyn/Syk activation. Cell Immunol..

[21] Takahashi M., Higuchi M., Makokha G.N., Matsuki H., Yoshita M., Tanaka Y., Fujii M. (2013). HTLV-1 Tax oncoprotein stimulates ROS production and apoptosis in T cells by interacting with USP10. Blood.

[22] Schank M., Zhao J., Wang L., Nguyen L.N.T., Zhang Y., Wu X.Y., Zhang J.Y., Jiang Y., Ning S.B., El Gazzar M. (2023). ROS-induced mitochondrial dysfunction in CD4 T cells from ART-controlled people living with HIV. Viruses.

[23] Stapleford K., Verburg S.G., Lelievre R.M., Westerveld M.J., Inkol J.M., Sun Y.L., Workenhe S.T. (2022). Viral-mediated activation and inhibition of programmed cell death. PLoS Pathog..

[24] El-Amine R., Germini D., Zalcharova V.V., Tsfasman T., Sheval E.V., Louzada R.A.N., Dupuy C., Bilhou-Nabera C., Hamade A., Najjar F. (2018). HIV-1 Tat protein induces DNA damage in human peripheral blood B-lymphocytes via mitochondrial ROS production. Redox Biol..

[25] Gimenez S., Hamrouni E., André S., Picard M., Soundaramourty C., Lozano C., Vincent T., Tran T.-A., Kundura L., Estaquier J. (2025). Monocytic reactive oxygen species-induced T cell apoptosis impairs cellular immune response to SARS-CoV-2 mRNA vaccine. J. Allergy Clin. Immunol..

[26] Kundura L., Gimenez S., Cezar R., André S., Younas M., Lin Y.L., Portalès P., Lozano C., Boulle C., Reynes J. (2022). Angiotensin II induces reactive oxygen species, DNA damage, and T-cell apoptosis in severe COVID-19. J. Allergy Clin. Immun..

[27] Rahmani Z., Huh K.W., Lasher R., Siddiqui A. (2000). Hepatitis B virus X protein colocalizes to mitochondria with a human voltage-dependent anion channel, HVDAC3, and alters its transmembrane potential. J. Virol..

[28] Piccoli C., Scrima R., Quarato G., D′Aprile A., Ripoli M., Lecce L., Boffoli D., Moradpour D., Capitanio N. (2007). Hepatitis C virus protein expression causes calcium-mediated mitochondrial bioenergetic dysfunction and nitro-oxidative stress. Hepatology.

[29] Lecoeur H., Borgne-Sanchez A., Chaloin O., El-Khoury R., Brabant M., Langonné A., Porceddu M., Brière J.J., Buron N., Rebouillat D. (2012). HIV-1 Tat protein directly induces mitochondrial membrane permeabilization and inactivates cytochrome oxidase. Cell Death Dis..

[30] Boonnate P., Kariya R., Okada S. (2023). Shikonin induces ROS-dependent apoptosis via mitochondria depolarization and ER stress in adult T cell leukemia/lymphoma. Antioxidants.

[31] Meuren L.M., Prestes E.B., Papa M.P., de Carvalho L.R.P., Mustafa Y.M., da Costa L.S., Da Poian A.T., Bozza M.T., Arruda L.B. (2022). Infection of endothelial cells by dengue virus induces ROS production by different sources affecting virus replication, cellular activation, death and vascular permeability. Front. Immunol..

[32] Zhang Y.A., Salinas I., Li J., Parra D., Bjork S., Xu Z., LaPatra S.E., Bartholomew J., Sunyer J.O. (2010). IgT, a primitive immunoglobulin class specialized in mucosal immunity. Nat. Immunol..

[33] Cui Z.W., Zhang X.Y., Wu C.S., Zhang Y.A., Zhou Y., Zhang X.J. (2020). Membrane IgM^+^ plasma cells in grass carp (*Ctenopharyngodon idella*): Insights into the conserved evolution of IgM^+^ plasma cells in vertebrates. Dev. Comp. Immunol..

[34] Lu T.Z., Liu X., Wu C.S., Ma Z.Y., Wang Y., Zhang Y.A., Zhang X.J. (2022). Molecular and functional analyses of the primordial costimulatory molecule CD80/86 and its receptors CD28 and CD152 (CTLA-4) in a teleost fish. Front. Immunol..

[35] Han X.Q., Pan Y.R., Zhong Y.Q., Tian T.T., Liu X., Zhang X.J., Zhang Y.A. (2024). Identification and functional analyses of CD4-1+ cells in grass carp (Ctenopharyngodon idella). Fish Shellfish Immunol..

[36] Liu J.X., Ren J.Q., Wang D., Wang Z., Ma X.Y., Zhou H. (2025). *Edwardsiella piscicida* promotes mitophagy to escape autophagy-mediated antibacterial defense in teleost monocytes/macrophages. Aquaculture.

[37] Lv M.Y., Wang Y.W., Yu J.Z., Kong Y.Y., Zhou H., Zhang A.Y., Wang X.Y. (2024). Grass carp IL-2 promotes neutrophil extracellular traps formation via inducing ROS production and autophagy in vitro. Fish Shellfish Immunol..

[38] Li J., Barreda D.R., Zhang Y.A., Boshra H., Gelman A.E., Lapatra S., Tort L., Sunyer J.O. (2006). B lymphocytes from early vertebrates have potent phagocytic and microbicidal abilities. Nat. Immunol..

[39] Wu C.S., Dai Y.S., Yuan G.L., Su J.G., Liu X.L. (2019). Immunomodulatory effects and induction of apoptosis by different molecular weight chitosan oligosaccharides in head kidney macrophages from blunt snout bream (*Megalobrama amblycephala*). Front. Immunol..

[40] Love M.I., Huber W., Anders S. (2014). Moderated estimation of fold change and dispersion for RNA-seq data with DESeq2. Genome Biol..

[41] Livak K.J., Schmittgen T.D. (2001). Analysis of relative gene expression data using real-time quantitative PCR and the 2(-Delta Delta C(T)) Method. Methods.

[42] Wei B., Cui Y., Huang Y.F., Liu H., Li L., Li M., Ruan K.C., Zhou Q., Wang C. (2015). Tom70 mediates sendai virus-induced apoptosis on mitochondria. J. Virol..

[43] Rao Y.L., Wan Q.Y., Su H., Xiao X., Liao Z.W., Ji J.F., Yang C.R., Lin L., Su J.G. (2018). ROS-induced HSP70 promotes cytoplasmic translocation of high-mobility group box 1b and stimulates antiviral autophagy in grass carp kidney cells. J. Biol. Chem..

[44] Xin L.S., Li C., Bai C.M., Wang C.M. (2018). Ostreid Herpesvirus-1 Infects Specific Hemocytes in Ark Clam. Scapharca Broughtonii Viruses.

[45] Wang Y.T., Liu W., Seah J.N., Lam C.S., Xiang J.H., Korzh V., Kwang J. (2002). White spot syndrome virus (WSSV) infects specific hemocytes of the shrimp. Dis. Aquat. Organ..

[46] Raja R., Sen G.C. (2022). The antiviral action of the RIG-I induced pathway of apoptosis (RIPA) is enhanced by its ability to degrade Otulin, which deubiquitinates IRF3. Cell Death Differ..

[47] Zhu H.C., Hou P.L., Chu F.Y., Li X.Y., Zhang W.J., Sun X.N., Liu Y., Zhao G.M., Gao Y.W., He D.C. (2024). PBLD promotes IRF3 mediated the type I interferon (IFN-I) response and apoptosis to inhibit viral replication. Cell Death Dis..

[48] Simpson D.S., Pang J., Weir A., Kong I.Y., Fritsch M., Rashidi M., Cooney J.P., Davidson K.C., Speir M., Djajawi T.M. (2022). Interferon-γ primes macrophages for pathogen ligand-induced killing via a caspase-8 and mitochondrial cell death pathway. Immunity.

[49] Nichols J.E., Niles J.A., Roberts N.J. (2001). Human lymphocyte apoptosis after exposure to influenza A virus. J. Virol..

[50] Roberts N.J. (2023). The enigma of lymphocyte apoptosis in the response to influenza virus infection. Viruses.

[51] Nichols J.E., Niles J.A., Fleming E.H., Roberts N.J. (2019). The role of cell surface expression of influenza virus neuraminidase in induction of human lymphocyte apoptosis. Virology.

