# Supplementary material for: GCRV-II Triggers B and T Lymphocyte Apoptosis via Mitochondrial ROS Pathway
Source: Viruses. 2025 Jun 30;17(7):930. doi: 10.3390/v17070930 (PMC12298793; doi:10.3390/v17070930)
Supplement: Supplementary file 1 [file viruses-17-00930-s001.zip › viruses-3669500-supplementary.pdf]

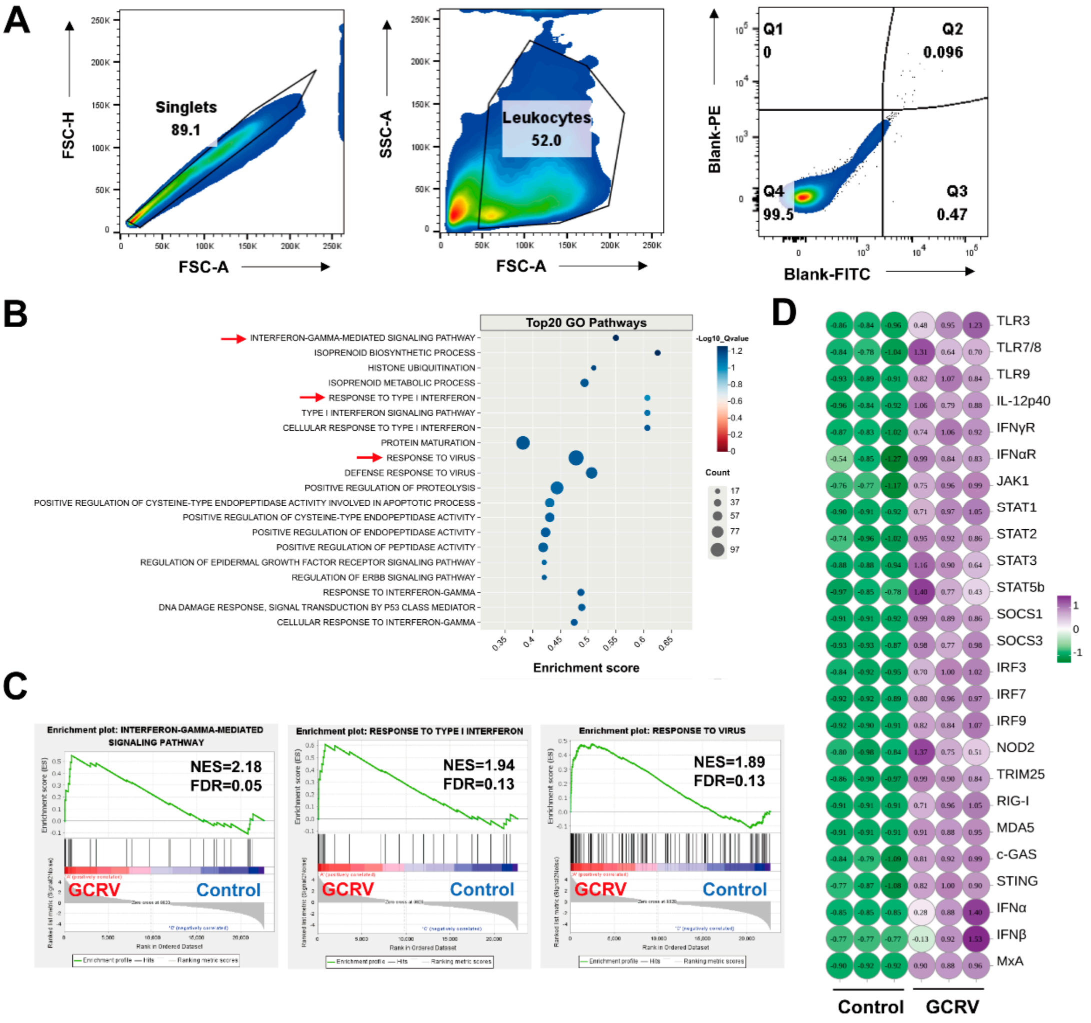

Figure S1. Flow cytometry gating strategy and antiviral immune pathways. (A) Flow cytometry gating strategy of leukocytes and blank control staining in PBLs, related to Figure 1D. (B) Top 20 GO pathways enriched for GCRV group by GSEA. The red arrows indicate the selected pathways in Figure S1C. (C) Significantly enriched gene sets related to antiviral interferon γ and type I interferon pathways in GCRV group at 36 h post infection (NES, normalized enrichment score; FDR, false discovery rate). (D) Heatmap analysis of phenotypic DEGs related to antiviral pathways (data adjusted $p < 0.01$, $|\log_2 FC| > 1$; violet, upregulated genes; green, downregulated genes). n = 3. PBLs, peripheral blood leukocytes; DEGs, differentially expressed genes; GSEA, gene-set enrichment analysis.

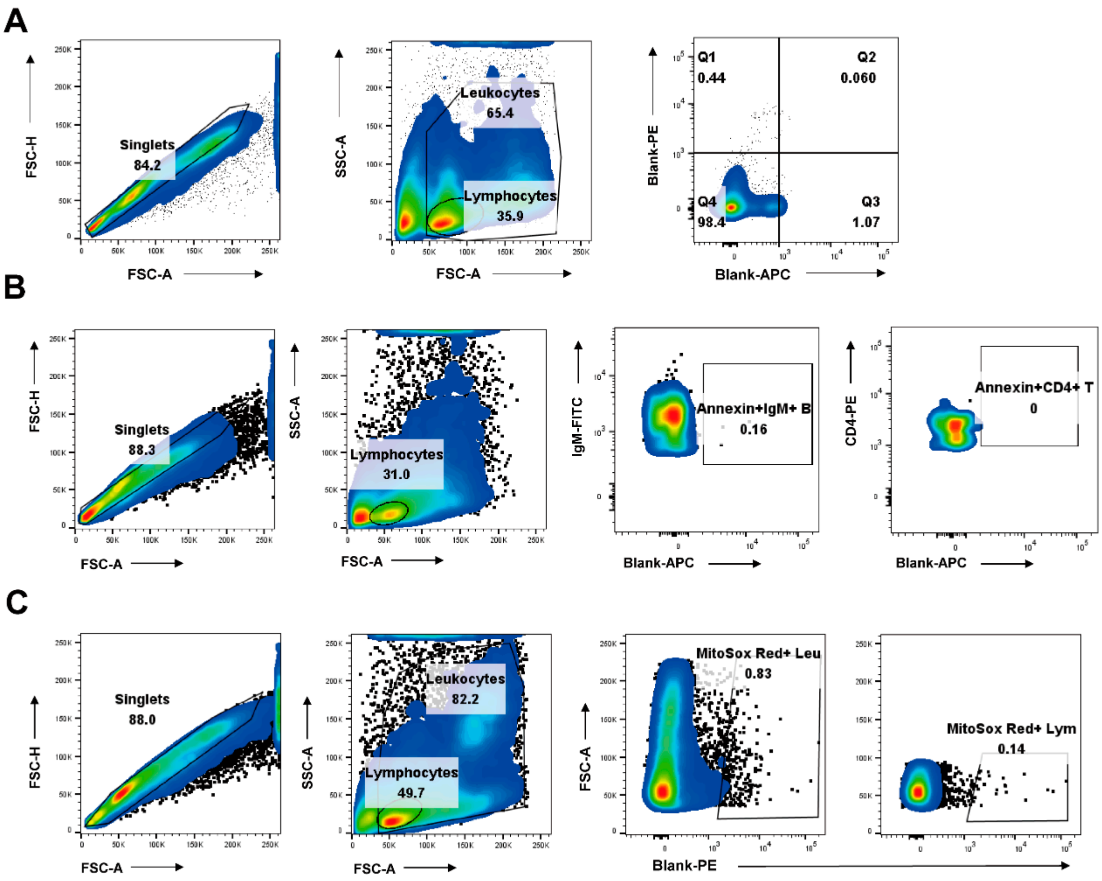

Figure S2. Flow cytometry gating strategy and blank control staining. (A) Flow cytometry gating strategy of leukocytes and lymphocytes, and blank control staining in PBLs, related to Figure 3A. (B) Flow cytometry gating strategy of B-T lymphocytes, and blank control staining in PBLs, related to Figure 4F and 5C. (C) Flow cytometry gating strategy of leukocytes and lymphocytes, and blank control staining in gated leukocytes and lymphocytes, related to Figure 5A. PBLs, peripheral blood leukocytes.
